# Kikuchi-Fujimoto disease: a case report and the evaluation of diagnostic procedures

**DOI:** 10.1186/s12903-019-0920-4

**Published:** 2019-10-21

**Authors:** Shenjie Xu, Weilian Sun, Jiamei Liu

**Affiliations:** Department of Oral Medicine, The Second Affiliated Hospital, School of Medicine, Zhejiang University, Hangzhou Zhejiang, 310009 People’s Republic of China

**Keywords:** Kikuchi-Fujimoto disease, Histiocytic necrotizing lymphadenitis, Diagnosis, Rare disease

## Abstract

**Background:**

Kikuchi-Fujimoto disease, known as histiocytic necrotizing lymphadenitis, is a benign, self-limiting and systemic disorder involving lymph nodes with unknown aetiology. First reported in Japan, there has been an increase in its incidence globally. Because of its non-specific clinical features, the disease can be easily mistaken for other forms of lymphadenitis with a high rate of misdiagnosis and mistreatment, posing a considerable challenge.

**Case presentation:**

A case of young Chinese woman with fever and cervical lymphadenopathy is presented. Physical examinations and imaging techniques were used to rule out common forms of lymphadenitis (e.g. cat-scratch disease and tuberculous lymphadenitis). Laboratory tests were then conducted to exclude others such as systemic lupus erythematosus and non-Hodgkin lymphoma. After Kikuchi-Fujimoto disease was identified, the patient was managed with symptomatic treatments. Our case was compared with relevant cases in the literature. A diagnostic flow chart was proposed to facilitate the diagnosis and treatment.

**Conclusions:**

With its shared clinical features, Kikuchi-Fujimoto disease can be mistaken for other forms of lymphadenitis. A combined use of medical imaging and laboratory tests is the effective way to avoid misdiagnosis.

## Background

Histiocytic necrotizing lymphadenitis (HNL) is a benign and self-limiting systemic disorder, involving lymph nodes with clinical manifestations of mild fever, superficial lymphadenopathy and rash among others. It is commonly known as Kikuchi-Fujimoto disease (KFD) as first reported in Japan by Kikuchi [1] and Fujimoto [2] in 1972. Due to its low incidence rate and non-specific clinical features, KFD has not been well understood. Although unidentified infectious agents and an autoimmune response have been considered as the prime causes, the KFD pathogenesis remains unclear [3]. Because of its shared clinical features, the disease can be easily mistaken for other forms of lymphadenitis with a misdiagnosis rate up to 40% [4]. Near half a century has passed, the difficulties remain in distinguishing KFD from other lymph node diseases and continue to pose significant risks to misdiagnosis and unnecessary treatment [5]. This is particularly so in the diagnosis and treatment of oral diseases as the posterior cervical chain is the most common location for KFD. Therefore, it become imperative to evaluate existing diagnostic procedures and to avoid misdiagnosis and mistreatment. This paper reports the case of a young Chinese woman with fever and painful cervical lymphadenopathy and presents a critical comparison with relevant cases in the literature. The paper also identifies and assesses practical steps for diagnosing and managing KFD.

## Case presentation

A 23-year-old female patient had mild fever and multiple swellings in the left neck and felt pain upon touch. She was generally healthy and fit. When asked, the patient denied the following abnormalities: headache, insomnia, anorexia and weight loss. Her condition had been previously considered as “lymphadenitis” in a local clinic, however it failed to abate even after 2 months’ treatment with anti-inflammatory drugs.

On physical examination, the face of the patient was found generally symmetrical, and the mouth could open normally. Several swelling masses were observed in the left neck with the diameter being about 1.5 cm, and were soft and tender to palpation. No obvious lesions were found in the oral cavity, except the left and right third molars were impacted. Magnetic resonance imaging (MRI) scans revealed multiple swollen lymph nodes in both sides of the neck, but most noticeably in the left. An ultrasound of her neck confirmed lymph node enlargements. Thus, she was admitted to the hospital for further diagnosis.

**Fig. 1:**
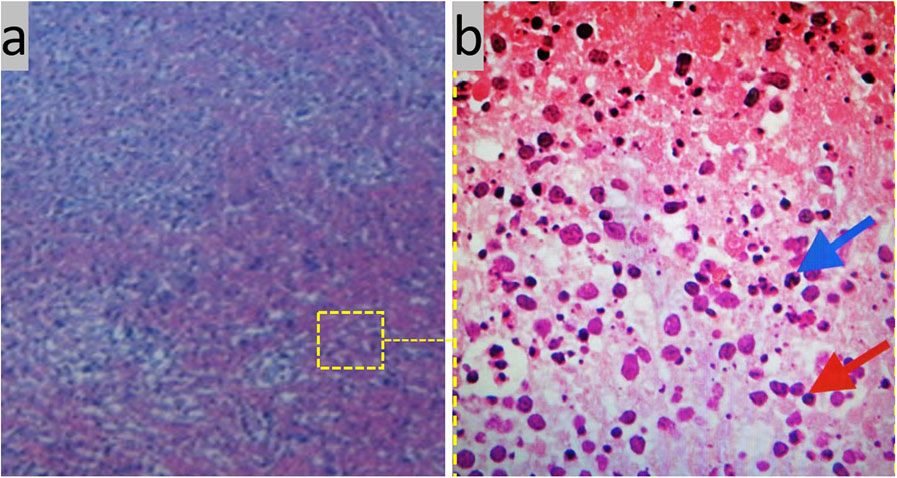
**a** Photomicrograph (magnified × 40) of paracortical area of the lymph node from the patient’s left neck; **b** an enlarged part, showing the characteristic features of HNL with multifocal coagulative necrosis: plentiful nuclear debris (arrowed in Blue), large mononuclear cells in the periphery (arrowed in RED), and no visible neutrophil in the necrotic area

With the patient’s consent, additional medical examinations and pathological analyses were carried out to determine the nature of the lesion. The results from chest X-ray filming, tuberculin test and nasopharyngeal biopsy were all negative, ruling out tuberculous lymphadenitis (TbL) and nasopharyngeal carcinoma. A cervical lymph node biopsy was conducted, revealing multifocal coagulative necrosis in the paracortical area, plentiful nuclear debris and large mononuclear cells in the periphery, as shown in Fig. 1. Immunohistochemical analysis showed the histocytes in the biopsy were both MPO (myeloperoxidase)-positive and CD68-positive, further pointing to histiocytic necrotizing lymphadenitis. Thus, the patient was kept at the hematology department in our hospital for further monitoring. During her stay in the hospital, she was reviewed daily and managed with symptomatic treatments to reduce her temperature and swellings. The patient was finally discharged in a month after a full recovery. There has been no sign of recurrence at 18-month follow-up.

## Discussion and conclusion

Kikuchi-Fujimoto disease (KFD) was first reported in 1972, however, its cause remains unknown. It is generally accepted that KFD is a non-neoplastic lymph node enlargement disease, a type of lymphoid reactive hyperplasia [3]. KFD was found mostly in young people [6] and with a very low recurrence rate (about 3%) [7]. Previously KFD was thought to be prevalent in women, but some studies indicate the disease could affect both sexes [8]. In the past, the disease was mostly noted in East Asia (e.g. Japan). However there has been a gradual increase in publication of KFD cases beyond East Asia.

To understand the disease and improve its diagnosis, it is crucial to have a comparative review of various aspects relating to the disease. We surveyed the literature on KFD and compared our case with some of those reported recently (see Table 1) on the key aspects, including sex, age, ethnic origin, symptom and treatment.

**Table 1:** A comparison with some cases reported in the literature

Year /country	Sex	Age	Ethnic	Symptom	Treatment
This case/China	F	23	China	Fever, Swelling	Antibiotics/ inpatient stay
2006/Turkey [9]	F	28	Turkey	Fever, Swelling, Weight loss	Ampicillin and prednisone
2008/UK [10]	M	55	N/S^a^	Fever, Weight loss, Anorexia	2 weeks’ inpatient stay
2013/Bangladesh [11]	F	17	N/S^a^	Fever, Swelling, Weight loss, Anorexia	Symptomatic treatment
2017/ Senegal [12]	F	33	Senegal	Fever, Swelling	Corticosteroid therapy
2018/USA [13]	F	19	Chinese	Fever, Sore Throat, Night Sweats	IV. ampicillin/sulbactam

^a^The ethnicity is not stated (N/S) explicitly, but can be considered implicitly as the locals

Table 1 clearly shows that these KFD cases have been reported from various continents, including other Asian countries (Turkey [9], Bangladesh [11] and Sri Lankan [14]), Europe (UK [10] and France [15]), Africa (Senegal [12]) and the USA [13], although the very first case out of East Asia was documented before 1982 [16]. Together with the fact that KFD has non-specific clinical features and that it can be easily confused with other diseases [13], the spread of KFD into other continents and other ethnic groups will certainly urge medical professionals to better understand and treat the disease.

To diagnose the disease promptly and accurately, it is essential to conduct an analysis of any abnormalities in the patient caused by the disease. The two of the most relevant aspects are clinical manifestations and pathological features.

**Clinical manifestations** of KFD are primarily fever and lymphadenopathy. The common symptoms can be summarized as follows: (a) fever with a body temperature fluctuated at 38 ~ 41 °C (some patients with mild chills), lasting about 4 to 6 weeks; (b) superficial lymph nodes, mainly in the neck, with a diameter of 0.5 to 3 cm; (c) congestive maculopapular rash, commonly in the trunk, limbs and cheeks with the skin being mildly edematous and itchy; (d) mild hepatosplenomegaly with around 0.5 to 2 cm enlargement of the liver. As these symptoms are not limited to this disease, KFD could be mistaken for others, e.g. malignant lymphoma. Thus more specific analyses must be carried out to avoid misdiagnosis and mistreatment.

**Pathological analysis** of the lymph node biopsy is frequently performed. The diseased tissues generally show extensive coagulative necrosis and histiocytosis in the cortical and paracortical regions of the lymph nodes (see Fig. 1). The histological features, such as clusters of plasma-like mononuclear cells with scattered nuclear debris and crescent-shaped tissue cells, are indistinguishable from those of lymphoma. So additional diagnostic procedures are required. For example, as immunohistochemical staining can reveal the presence of MPO-positive and CD68-positive cells [7], such a pathological tool would be useful for both diagnostic and differential diagnosis.

### Medical imaging

In comparison with pathological analysis such as biopsy, medical imaging is much quicker and less invasive. Magnetic resonance image and ultrasound scan have been used in our case to confirm lymph node enlargement. Other imaging technologies such as positron emission tomography/computed tomography have also been exploited [17], however they can provide useful evidence, but not a definitive diagnosis.

### Diagnosis and differential diagnosis

As discussed above, the main symptoms for KFD are un-explained fever and lymphadenopathy. Thus, the following observations are particularly worth noting: (a) long-term fever even after antibiotic treatment; (b) large superficial lymph nodes, but little or no sign of hepatosplenomegaly and (c) negative results from blood test and bone marrow culture. Due to non-specific clinical manifestations, the disease can be misdiagnosed as other disorders, such as tumors (e.g. Non-Hodgkin’s lymphoma) or infectious diseases (e.g. TbL or cat scratch disease [18]) or concurrent systemic lupus erythematosus (SLE). A recent study [19] reported 12% KFD patients had a history of SLE. A comparison of KFD with some related disorders is shown in Table 2, listing different characteristics for each of the disorders.

**Table 2:** A comparison of KFD with some lymphadenitis diseases

	Pain	Fever	Self-limiting	Distinguishing features
KFD (HNL)	Some	Irregular	Yes	Coagulative necrosis, nuclear debris
NHL	Yes	Relapsing	No	Malignant hyperplasia, monoclonal lymphocytes
TbL	Some	Intermittent	No	Caseous necrosis, tuberculous granuloma
CSD	Yes	Irregular	Yes	Central necrosis, granulomatous inflammation
SLE	Some	Irregular	No	Hematoxylin bodies, abundance of plasma cells

*HNL* Histiocytic necrotizing lymphadenitis, *NHL* Non-Hodgkin’s Lymphoma, *TbL* Tuberculous lymphadenitis, *CSD* Cat-scratch disease, *SLE* Systemic lupus erythematosus

Table 2 shows that fever exists in all these listed disorders, so cannot be used to distinguish KFD from others. However, detailed temperature charts may be helpful. For instance, KFD patients, generally, show an irregular fever pattern while patients with non-Hodgkin’s lymphoma (NHL) have relapsing fever and those with TbL experience intermittent fever. Although pain is felt in some of the listed disorders, but not always complained of by the patients. The sensation of pain is rather subjective and thus cannot be used for differential diagnosis. The self-limiting factor is well noteworthy during the management of the symptoms, however, it is no practical use in terms of diagnosis.

**Fig. 2:**
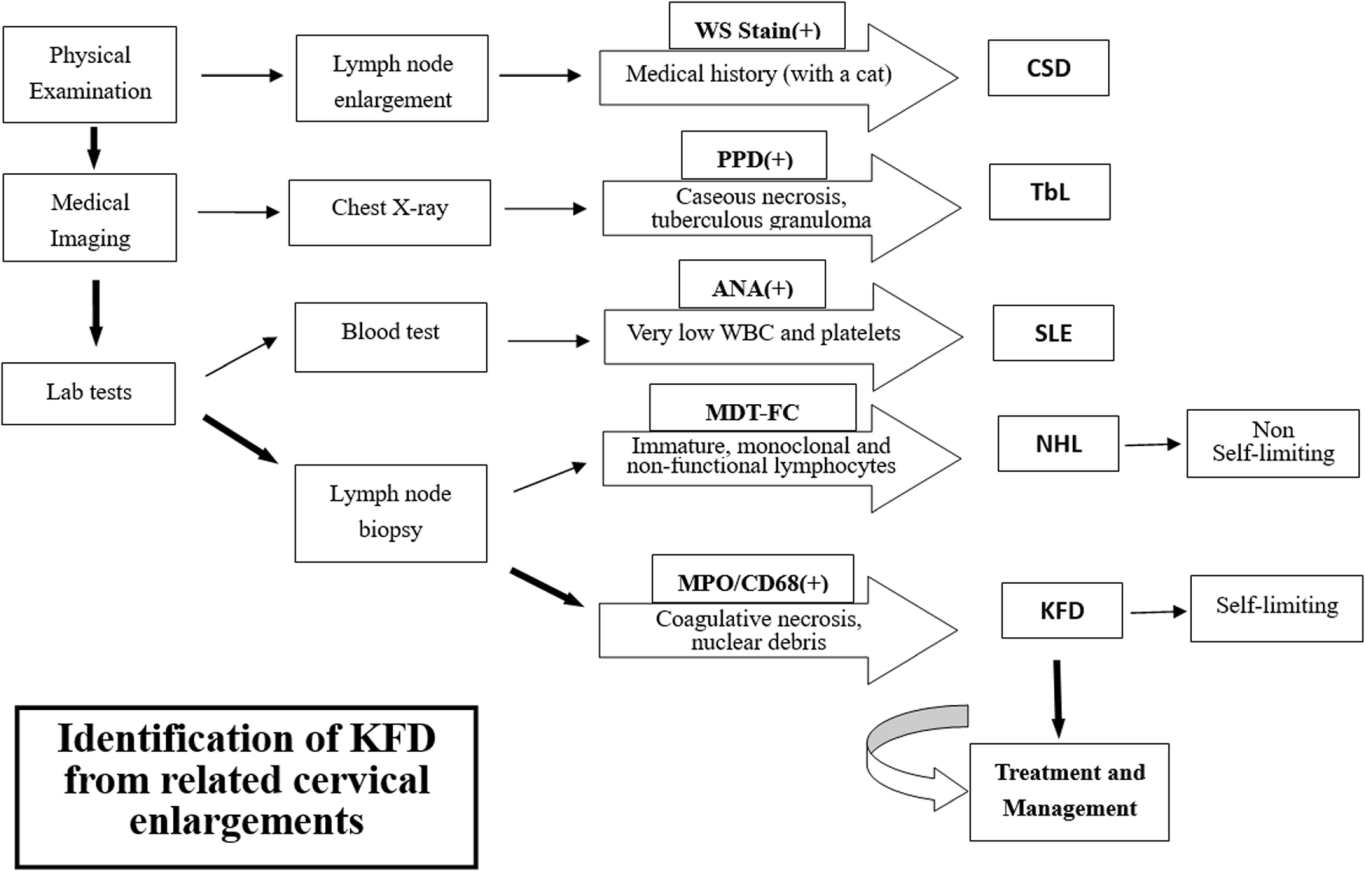
A flow chart for identifying KFD (Kikuchi-Fujimoto Disease) from other forms of cervical lymphadenitis. WS Stain: Warthin-Starry stain; PPD test: Purified protein derivative test; ANA: Anti-nuclear antibody; MDT-FC: Molecular diagnostic test and flow cytometry; MPO/CD68: myeloperoxidase/cluster of differentiation 68 protein. CSD: Cat-scratch disease; TbL: Tuberculous lymphadenitis; SLE: Systemic lupus erythematosus; NHL: Non-Hodgkin’s Lymphoma. KFD: Kikuchi-Fujimoto disease

Although histopathological features are not always the same among all these disorders, they do display certain characteristics useful for diagnosis. KFD and NHL share certain histopathological features, such as proliferation of immunoblasts and plasmacytoid dendritic cells at the edges of necrotic foci [20]. Immuno-staining would be helpful as the positivity of histiocytes for myeloperoxidase can be used to exclude T-cell lymphomas and can offer useful clues regarding infectious agents. For instance, the positivity of PPD test can certainly consolidate the diagnosis of tuberculosis while the finding of *Bartonella henselae* by Warthin–Starry stain would almost certainly confirm the case for CSD (as illustrated in Fig. 2). As to systemic lupus erythematosus (SLE) lymphadenopathy which is the hardest one to differentiate from KFD, the detection of hematoxylin bodies should add further support to the diagnosis of SLE over KFD.

Clearly, the diagnosis of KFD can be difficult and its differential diagnosis is more demanding. To aid medical practitioners, in particular, non-specialists to identify this rare disorder, a diagnostic flow chat is proposed in Fig. 2. Once a cervical lymphadenopathy is presented, appropriate medical assessments should be carried out. Physical examinations and imaging techniques can be used to rule out certain forms of lymphadenitis (e.g. CSD and TbL). With further laboratory tests, after excluding serious lymph node diseases such as SLE and NHL, KFD can be confirmed, treated and managed.

### Treatment and management

Clinically, patients with unexplained fever should be carefully examined and closely monitored regarding any change with lymph nodes. If no improvement is observed in a week or so after the treatment with antibiotics, medical professionals should consider the possibility of KFD by conducting a lymph node biopsy to determine pathological features. Once KFD is confirmed and malignant lymphoma or other lymphatic diseases (such as TbL or CSD) are ruled out, appropriate steps are taken to facilitate the relief of the symptoms.

KFD is a subacute disease, mostly lasting 1 to 3 months, but some persisting for up to 1 year [8]. There is no universally agreed treatment plan as each case could be somehow different. The primary treatment of KFD is to manage the disease by supporting the patient mentally and physically to speed-up the relief of the symptoms. Table 1 lists some successful treatments to relieve the KFD symptoms. Antibiotics are not effective, however, their use in this case [13] is to avoid potential bacterial infections. The use of hormone-related drugs can relieve the symptoms and shorten the course of the disease. Prednisone was used as an effective treatment for pregnant women [9]. Generally, KFD is a benign and self-limiting disorder, so a short inpatient stay with symptomatic treatment (as in our case) generally suffice. However, for patients with significant systemic symptoms (e.g. multiple organ systems failure or immune system dysfunction), appropriate drugs such as non-steroidal anti-inflammatory drugs (NSAIDs), hormones and immunosuppressants should be seriously considered to manage the symptoms while further specific diagnosis are to be carried out.

In summary, Kikuchi-Fujimoto disease is a rare disorder found mostly in young women of oriental origin with a slow spread to other ethnicities. Although benign and self-limiting in most cases, it can confuse medical professionals with other life-threatening diseases. Early and reliable diagnosis of the disease using imaging technologies and laboratory tests and appropriate management of the symptoms are uppermost to avoid misdiagnosis and mistreatment. The diagnostic flow chart illustrated in the paper should be helpful to medical professionals, particularly to non-specialists including oral health practitioners.

## Data Availability

The data used during the study are available from the corresponding author on reasonable request.

## Abbreviations

CSD: Cat-scratch disease
HNL: Histiocytic necrotizing lymphadenitis
KFD: Kikuchi-Fujimoto disease
NHL: Non-Hodgkin’s lymphoma
SLE: Systemic lupus erythematosus
TbL: Tuberculous lymphadenitis
